# Study on Demulsification-Flocculation Mechanism of Oil-Water Emulsion in Produced Water from Alkali/Surfactant/Polymer Flooding

**DOI:** 10.3390/polym11030395

**Published:** 2019-02-28

**Authors:** Bin Huang, Xiaohui Li, Wei Zhang, Cheng Fu, Ying Wang, Siqiang Fu

**Affiliations:** 1 The Key Laboratory of Enhanced Oil and Gas Recovery of Educational Ministry, Northeast Petroleum University, Daqing 163318, China; huangbin111@163.com (B.H.); lixiaohuibusiness@163.com (X.L.); 2 Shandong Key Laboratory of Oil-Gas Storage and Transportation Safety, College of Pipeline and Civil Engineering, China University of Petroleum, Qingdao 266580, China; 15776541967@163.com; 3 Post-Doctoral Research Station of Daqing Oilfield, Daqing 163458, China; 4 Aramco Asia, Beijing 100102, China; ying.wang.1@aramcoasia.com; 5 No. 1 Oil Production Plant in Daqing Oilfield, Daqing 163001, China; fusiqiang@petrochina.com.cn

**Keywords:** Alkali/Surfactant/Polymer flooding, produced water, oil-water emulsion, demulsification, flocculation

## Abstract

The issue of pipeline scaling and oil-water separation caused by treating produced water in Alkali/Surfactant/Polymer (ASP) flooding greatly limits the wide use of ASP flooding technology. Therefore, this study of the demulsification-flocculation mechanism of oil-water emulsion in ASP flooding produced water is of great importance for ASP produced water treatment and its application. In this paper, the demulsification-flocculation mechanism of produced water is studied by simulating the changes in oil-water interfacial tension, Zeta potential and the size of oil droplets of produced water with an added demulsifier or flocculent by laboratory experiments. The results show that the demulsifier molecules can be adsorbed onto the oil droplets and replace the surfactant absorbed on the surface of oil droplets, reducing interfacial tension and weakening interfacial film strength, resulting in decreased stability of the oil droplets. The demulsifier can also neutralize the negative charge on the surface of oil droplets and reduce the electrostatic repulsion between them which will be beneficial for the accumulation of oil droplets. The flocculent after demulsification of oil droplets by charge neutralization, adsorption bridging, and sweeping all functions together. Thus, the oil droplets form aggregates and the synthetic action by the demulsifier and the flocculent causes the oil drop film to break up and oil droplet coalescence occurs to separate oil water.

## 1. Introduction

After years of development, most oil fields in China have now entered the late stage of high water cut development. Daqing Oilfield was the first in the world to commercialize the technology of Alkali/Surfactant/Polymer (ASP) flooding in 2014. ASP flooding has proved to be effective in improving oil recovery by 20% compared with water flooding. It has seen industrial application in the Daqing Oilfield [1].

Although ASP flooding technology can greatly increase field output and oil recovery compared with water flooding, it also brings severe challenges to oilfield surface engineering. Compared with the new methods of development with less pollution such as microbial flooding and nano-fluid flooding [2,3,4], pipeline scaling is a serious issue causing pipes to become easily blocked due to the existence of oil displacement agents. It has a serious influence on production and even safety accidents [5,6,7]. At the same time, the existence of residual chemical agents in the ASP flooding produced water will lead to complications in the produced water [8,9]. The produced water has a high salinity, high degree of emulsification, strong interfacial film strength and high content of small oil droplets [10,11,12]. As a result, it is difficult to separate oil and water which greatly limits commercialized ASP flooding technology.

In order to form an ultra-low oil-water interfacial tension during the ASP flooding process, a surfactant was added. The flow with low interfacial tension can pass easily through the porous medium, resulting in an improvement in oil recovery [13]. However, the addition of a surfactant not only enhances oil recovery, but also inevitably causes the serious emulsification of the ASP flooding produced water, which increases the stability of the oil droplets, resulting in difficulty treating the produced water.

Adequate mixing and the presence of a surface-active agent are two vital factors that lead to the emulsion formation when the oil and water phases are brought together [14]. The oil droplets rupture under the action of viscous shear stress. The alkali and surfactant reduce the interfacial tension greatly, which reduces the stability of the oil droplets. The polymers increase the emulsion stability by increasing water phase viscosity, decreasing the rate of raising the oil droplets, and higher polymer concentration results in higher interfacial film strength. The alkali enhances the stability of emulsion by reacting with acidic substances in crude oil to form a surfactant. The surfactant can adsorb onto the oil-water interface, which reduces the interfacial tension and increases the strength of the interface membrane. On the one hand, the capillary force that promotes the drainage of the liquid film and the fluidity at the oil-water interface is reduced. On the other hand, the electrostatic repulsion on both sides of the liquid film is reduced. The combined effect acts as a hindrance in the coalescence of oil beads. Under the interaction of the alkali, surfactant and polymer, the system is emulsified and forms the O/W emulsion [15,16].

The emulsion in ASP flooding produced water causes difficulties in its treatment. Many conventional processes have been used to treat produced water in oilfields, including gravity separation [17], floatation [18,19], demulsification [20,21], membrane separation [22], air flotation [23], adsorptive separation [24] and biotechnology [25]. However, only a few studies have reported the treatment of produced water from ASP flooding. Deng et al. studied the influence of an oil displacement agent on the stability of oil droplets in ASP flooding water and the influence of different demulsifiers and flocculants on the stability of oil droplets [26,27]. It was found that the effect of the oil-soluble demulsifier was better than that of water-soluble demulsifier. Oil-soluble demulsifiers can reduce the oil-water interfacial tension and Zeta potential on the surface of oil droplets. Therefore, it can increase the oil droplet size and facilitate the coalescence of oil droplets. Wang et al. studied the influence of alkali, polymer and surfactant on oil-water separation of simulated produced water from ASP flooding [28]. The results showed that the simulated produced water from ASP flooding can be successfully treated using a leaching solution of alkaline white mud. Due to the significant effect of the white mud on the treatment of produced water, Gao et al. [29] further studied this phenomenon. They evaluated the efficiency of removing emulsified oil by metallic hydroxides were in-situ generated (IGMHs) from simulated ASP flooding produced water and found the oil removal rate was up to 99%. They proved the economic viability of treatment process recovery of oil from oily wastewater. Zhang et al. [30] investigated the removal efficiency of PTFE film on the main pollutants of ASP produced water and proved that PTFE film also achieves a good treatment effect of produced water. Miao et al. [31] studied the effect of asphaltene on oil-water interface properties. The study showed that asphaltene aggregated to form film on the oil-water interface. The effluent velocity of the liquid film was reduced and the stability of produced water was improved.

Although these methods have a good performance in the treatment of ASP flooding produced water, they are limited to laboratory experiments and cannot be commercialized. It is difficult to achieve the reinjection standard by only using a demulsifier or flocculant alone to treat the produced water from ASP flooding. Therefore, we consider using a demulsification-flocculation method to improve the treatment effect of produced water. The produced water is firstly demulsified and then flocculated. In order to verify the feasibility of treatment of produced water by the demulsification-flocculation method, the demulsification-flocculation mechanism of oil-water emulsion in ASP flooding produced water must be fully understood. In this study, the effect of the demulsification-flocculation mechanism of oil-water emulsion from ASP flooding produced water was investigated with regards to interfacial tension, Zeta potential, and median size and microscopic morphology of oil droplets.

## 2. Materials and Experimental Methods

### 2.1. Experimental Materials and Instruments

Table 1 shows the experimental materials and sources used.

Distilled water was used in the experiments. The crude oil had a moisture content of less than 0.5%, a viscosity of 60 mPa·s, and a density of 860 kg/m^3^ at a temperature of 45 °C. The crude oil was provided by No. 1 Oil Production Plant in Daqing Oilfield. The polyacrylamide, hydrolyzed polyacrylamide (HPAM), with 300 × 10^4^ molecular weight and 25–30% hydrolysis degree, was from the Daqing Oilfield Production Engineering Research Institute. The surfactant with 50% effective content was alkyl benzene sulfonate, ORS-41. This was provided by the No. 1 Oil Production Plant in Daqing Oilfield. The alkali was reagent grade NaOH. The oil-soluble demulsifiers DYRPR-1625 and DPR-1870 and the organic flocculents cationic polyacrylamide (CPAM) and D2N-1650 provided by the No. 1 Oil Production Plant in Daqing Oilfield were used in the experiments.

Information regarding the experimental instruments is shown in Table 2.

### 2.2. Experimental Procedure

*Preparation of simulated mineralized water*. According to the underground water and discharge water qualities in the Daqing oilfield, mineralized water was prepared, and the salts contained in the mineralized water were as follows (mg/L): NaCl 1523, NaHCO_3_ 2820, Na_2_CO_3_ 168.7, Na_2_SO_4_ 10.5, CaCl_2_ 56.9, MgCl_2_·6H_2_O 35.5. The degree of mineralization of the mineralized water was 4614.6 mg/L.

*Preparation of simulated ASP flooding produced water*. The simulated ASP flooding produced water with a polymer (HPAM) concentration of 400 mg/L, surfactant (ORS-41) concentration of 200 mg/L, and alkali (NaOH) concentration of 800 mg/L.

Treated with xylene, the 1% demulsifier was used as a solvent. The demulsifier solution was added into the produced water, stirred at a speed of 200 r/min for 2 min and settled in the 45 °C water bath for 4 h. The demulsifier, ZY, was a compound of DYRPR-1625 and DYRPR-1870 at a ratio of 3:1.

The flocculent was added into the produced water, stirred for 2 min at 200 r/min and settled in the 45 °C water bath for 4 h. The flocculent, WS, was a compound of CPAM and D2N-1650 at a ratio of 3:1.

Interfacial tension, Zeta potential and oil droplet size were measured after adding demulsifier and flocculent.

### 2.3. Experimental Methods

The experimental methods for the preparation of simulated ASP flooding produced water, determination of the interfacial tension, Zeta potential, oil droplet size and observation of the microscopic morphology of oil droplets were as follows:

*Preparation of simulated ASP flooding produced water*. The mineralized water was used to prepare simulated produced water from ASP flooding in the lab and the process is as follows: 200 g of mineralized water with 0.1% surfactant and 200 g of crude oil were added to a 500 mL jar. The mixture was then heated to 45 °C in a water bath for 60 min. Then, the mixture was emulsified for 5 min at 20,000 rpm with a T25 emulsifier (IKA Company, Guangzhou, China) to obtain a 50% oil-water mixture. Next, 0.4 g of the 50% oil-in-water emulsion was added to 99.4 mL mineralized water with different concentrations of oil displacement agents, and the mineralized water was shaken to produce simulated produced water with an oil concentration of 2000 mg/L.

*Determination of the oil-water interfacial tension*. The oil-water interfacial tension was determined by a spinning drop method and measured by the interfacial tension meter after 60 min. The rotation rate was 8500 r/min. The temperature was 45 °C. The oil-water interfacial tension was obtained by Equation (1):

(1) $$\gamma =1.233\times 10^{3}\times \Delta \rho \times {\left(Kdsv\right)}^{3}{\left(6000/S\right)}^{-2},$$

where the Δρ is density between phases, kg/m^3^; K is the amplification factor; dsv is droplet width, m; and S is speed, r/min.

*Determination of the Zeta potential*. The Zeta potential of the produced water from the ASP flooding was conducted on a Micro electrophoresis apparatus. Firstly, 100 mL of the simulated produced water with the initial oil concentration of 1000 mg/L at different demulsifiers and flocculents were allowed to settle for 4 h at 45 °C. Then, a 5–10 mL sample was taken out of the jar with a syringe and added to the test utensil of the Micro electrophoresis apparatus for Zeta potential measurement. All measurements were repeated five times. The average values of these readings were reported.

*Determination of the oil droplet size*. The size of the oil droplets was determined by a mastersizer. All experiments were repeated for 3~5 times. The average values of these readings were reported.

*Micrograph observations*. A drop of produced water was placed on a glass holder held on the platform of a biological microscope and then observed and recorded on a camera. The magnification used was 10 × 40.

## 3. Results

### 3.1. The Effect of Demulsifier on Interfacial Tension

**Figure 1:**
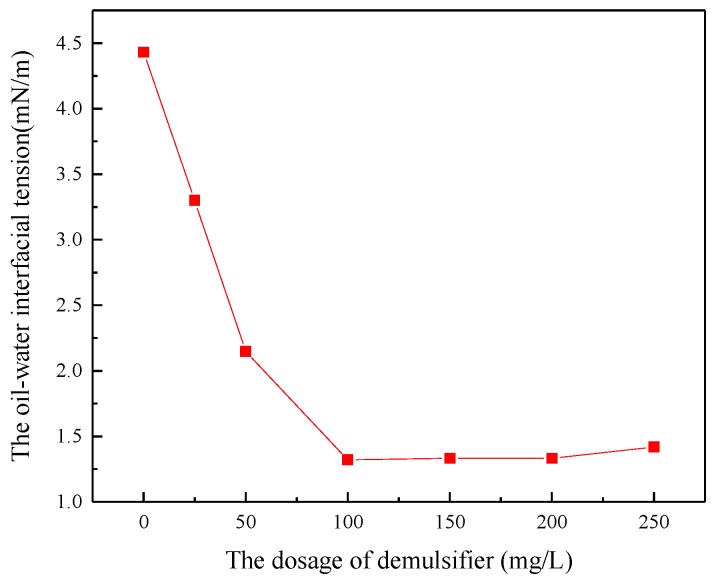
The effect of demulsifier on interfacial tension.

The simulated ASP flooding produced water with 400 mg/L of the polymer concentration, 200 mg/L of the surfactant concentration and 800 mg/L of the NaOH concentration was prepared and settled in a 45 °C water bath for 4 h to study the effect on oil-water interfacial tension in Figure 1. For the emulsion of ASP flooding produced water without adding a demulsifier, the effect of NaOH on interfacial tension of the system was mainly achieved by the surface-active components formed by the reaction with the acidic components and it had no obvious influence by itself on the interfacial tension. Surfactants had a great influence on the interfacial tension of the system, while the polymer had no effect on the interfacial tension. After adding the demulsifier, it can be observed that the demulsifier can reduce the oil-water interfacial tension. When the dosage of demulsifier increased from 0 to 100 mg/L, the interfacial tension of oil-water decreased from 4.425 mN/m to 1.325 mN/m, and then stabilized even with further addition from 100 mg/L to 200 mg/L. When the dosage exceeded 200 mg/L, the interfacial tension began to increase gradually. This is due to the fact that the very active demulsifier molecules can adsorb to the oil-water interface, thus reducing the oil-water interfacial tension. The adsorption of demulsifier molecules on the oil-water interface increased gradually with the increasing dosage of demulsifier. When the concentration reached 100 mg/L, the adsorption of demulsifier molecules on the interface reached saturation. Therefore, the oil-water interfacial tension decreased in concentration when the concentration of demulsifier further increased. Then, when the concentration of the demulsifier was higher than its critical micelle concentration (CMC), the demulsifier molecules began to aggregate into clusters to form micelles which caused the oil-water interfacial tension. In this case, the effect of demulsification and stabilization was reduced.

### 3.2. The Effect of Demulsifier on Zeta Potential

**Figure 2:**
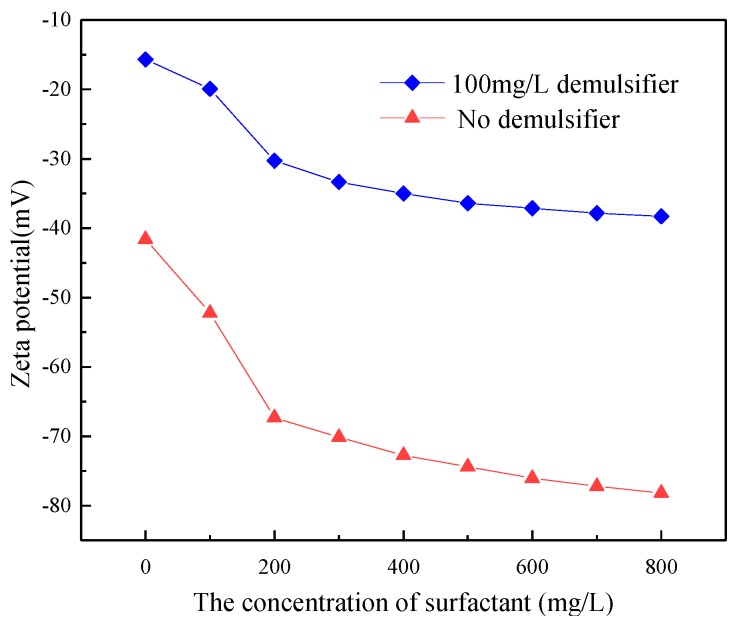
The effect of demulsifier on Zeta potential.

The simulated ASP system with 400 mg/L of the polymer, 800 mg/L of NaOH concentration and 0~600 mg/L of the surfactant concentrations were prepared. Then, 100 mg/L of demulsifier ZY was added into the produced water. The ASP system was settled in a 45 °C water bath for 4 h to study the effect of the demulsifier on the Zeta of potential surfactants with different concentrations, as shown in Figure 2. For the emulsion of ASP flooding produced water without adding demulsifier, the surfactants were absorbed onto the oil droplets with its non-polar head and polar head group extending in water. The Zeta potential on the surface of the oil droplet decreases quickly because the negative charges on the surface of the oil droplet increased. NaOH reacted with acidic components of crude oil and produced some surface-active components which adsorbed on the surface of oil droplets to reduce the Zeta potential. With increasing NaOH, the acid components were reacted completely. The electric double layer was compressed by the Na^+^ at the oil-water interface, resulting in the Zeta potential increasing on the surface of the oil droplet. The Zeta potential increased as a result of the above two combined effects. The polymer had a limited effect on the Zeta potential and stability of the system because of its low surface activity. After adding demulsifier, the surfactant can significantly improve the stability of the system. A higher concentration of the surfactant results in better stability of the system [32,33]. Compared to the system with no demulsifier, with 100 mg/L of demulsifier the absolute value of the Zeta potential on the surface of oil droplets decreased significantly at the same surfactant concentration and the stability of the system was changed from stable to unstable. The Zeta potential of the oil droplets changed from −41.234 mV to −15.547 mV because the 100 mg/L of demulsifier was added when the surfactant concentration is 0. The Zeta potential of the oil droplet was 62.3%. The Zeta potential of the oil droplet was changed from −67.125 mV to −30.625 mV when 100 mg/L of demulsifier was added to 200 mg/L of the surfactant solution. The absolute value of the Zeta potential of the oil droplet changed from 54.9% to 51.8% when the concentration of surfactant increased to 400 mg/L, while the absolute value of Zeta potential was stabilized with increasing surfactant concentration. The effect of demulsifier on the Zeta potential of oil droplets decreases gradually with increasing surfactant concentration.

### 3.3. The Effect of Demulsifier on the Size of Oil Droplets

**Figure 3:**
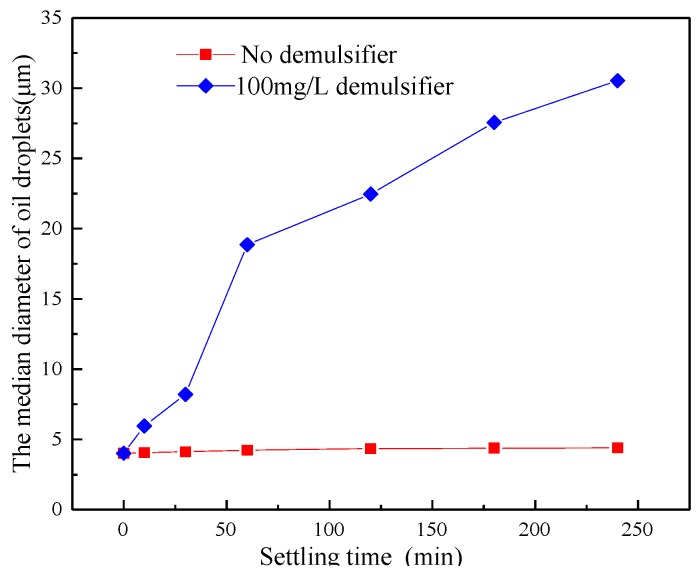
The effect of demulsifier on the median diameter of oil droplets.

**Figure 4:**
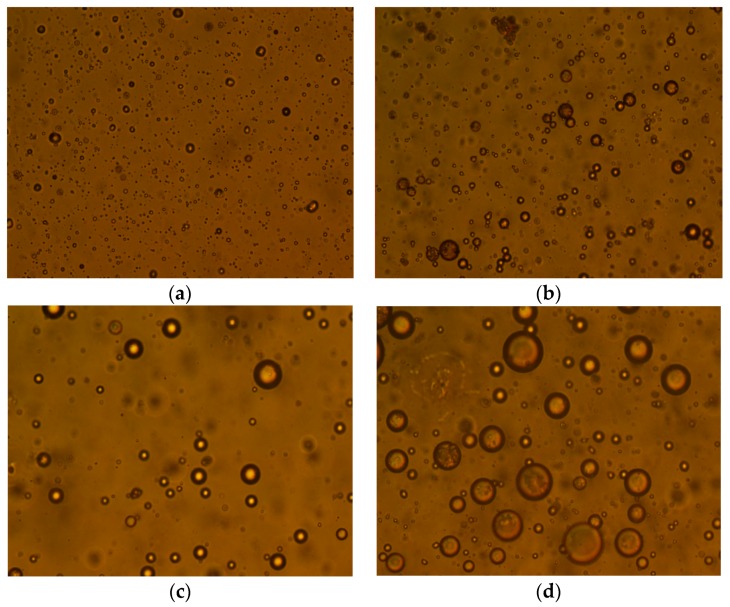
The effect of demulsifier on the size of oil droplets. (**a**) Settlement without demulsifier for 0 min, (**b**) settlement without demulsifier for 120 min, (**c**) settlement with demulsifier for 0 min, (**d**) settlement with demulsifier for 120 min.

The simulated ASP system with 400 mg/L of the polymer concentration, 800 mg/L of NaOH and 200 mg/L of the surfactant were prepared. Then, 100 mg/L of demulsifier ZY was added into the produced water. The ASP system was settled in a 45 °C water bath for 2 h to study the effect of the demulsifier on the size of the oil droplet, as shown in Figure 3. For the emulsion of ASP flooding produced water without adding demulsifier, NaOH and surfactant prevented oil droplets from coalescing but the polymer helped the oil droplets to coalesce. This is because the NaOH can react with the acidic components of crude oil and produce some surface-active components. Hence the oil-water interfacial properties changed, which can cause the oil droplets to become more stable. The size of oil droplets decreased with increasing surfactant concentration, which was due to the surfactant increasing the Zeta potential on the surface of the oil droplets and the electrostatic repulsion between oil droplets, resulting in a reduced probability of collision between oil droplets. The polymer had a dual function on the size of the oil droplet. On one hand, the polymer molecule had a bridging and flocculation effect for low polymer concentrations and compressed the double electric layer on the surface of the droplets, which is helpful for the aggregation and coalescence of oil droplets. On the other hand, the polymer molecules covered the surface of the oil droplets creating a steric hindrance effect with a high concentration, which was not conducive to the coalescence of oil droplets. After adding demulsifier, the demulsifier molecules adsorbed onto the surface of the oil droplets, resulting in a weakening of the steric hindrance effect of the polymer molecules. Therefore, the polymer molecules in the produced water were beneficial in the separation of emulsions. The demulsifier can significantly increase the median diameter of oil droplets. The median diameter of oil droplets increased from 4.02 μm to 4.40 μm when settled for 4 h without a demulsifier. The small increase of oil droplets indicated that the ASP flooding produced water was very stable. However, when settled for 2 h with added demulsifier ZY, the median diameter of oil droplets increased from 4.02 μm to 30.54 μm. The significant increase in the median diameter of oil droplets showed that the oil droplets in the ASP flooding produced water had become unstable after adding demulsifier. In order to further study the effect of demulsifier on the size of oil droplets, the shapes of oil droplets in the simulated produced water in different conditions were investigated with a microscope. The results are shown in Figure 4. The magnification used was 10 × 40. The size of the oil droplets was basically stable without demulsifier and settling for 2 h. The size of the oil droplets increased significantly after adding demulsifier and settled for 60 min. By increasing the settling time, the size of oil droplets increased gradually.

By summarizing the above experimental results, the interaction of ASP with the added demulsifiers can be found. The effect of NaOH on the stability of produced water was mainly achieved by the surface-active components formed from the reaction with the acidic components of crude oil, which produces some surface-active components which adsorbed on the surface of the oil droplets. During demulsification, the demulsifier molecules can be adsorbed onto the oil droplets, replacing the surfactant absorbed on the surface of oil droplets and resulting in a weakening of the steric hindrance effect of the polymer molecules. The processes reduced interfacial tension and weakened interfacial film strength, so that the stability of oil droplets was decreased. At the same time, the demulsifier can also neutralize the negative charge on the surface of the oil droplet, reducing the electrostatic repulsion between the oil droplets and aiding the accumulation of oil droplets.

### 3.4. The Effect of Flocculant on Interfacial Tension

**Figure 5:**
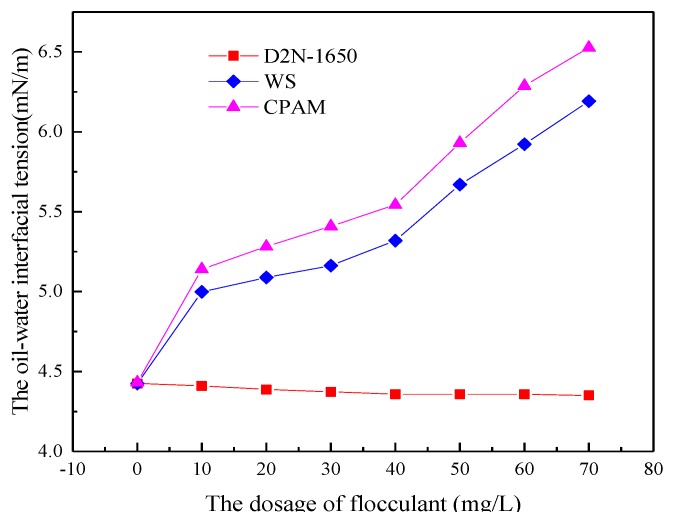
The effect of flocculant on interfacial tension.

The simulated ASP flooding produced water with 400 mg/L of polymer, 200 mg/L of surfactant concentration and 800 mg/L of NaOH concentration was prepared and settled in a water bath temperature of 45 °C for 4 h to study the effect of flocculant WS on oil-water interfacial tension. The effects of CPAM and D2N-1650 on oil-water interfacial tension were studied respectively under the same experimental conditions, as shown in Figure 5. The flocculant WS can increase the oil-water interfacial tension. When the concentration of flocculant increased from 0 to 70 mg/L, the interfacial tension of oil-water increased from 4.425 mN/m to 6.153 mN/m. The flocculant WS can only slightly increase oil-water interfacial tension, and its ability to do so is a result of the CPAM in the flocculant WS. With increasing concentration of the flocculant WS, the concentration of polyacrylamide in the system increased. Therefore, the viscosity of the system increased and the transportation and adsorption of surfactant on the oil-water interface was influenced. Thus, the amount of surfactant reaching the oil-water interface decreased, resulting in an increase in oil-water interfacial tension. However, the hindrance was weak, so the increase of oil-water interfacial tension was limited. The flocculant CPAM had no effect on the oil-water interfacial tension [34]. Compared to flocculant D2N-1650, the flocculent CPAM played a larger role in reducing the oil-water interfacial tension. Finally, the concentration of flocculant WS can increase the oil-water interfacial tension.

### 3.5. The Effect of Flocculant on Zeta Potential

**Figure 6:**
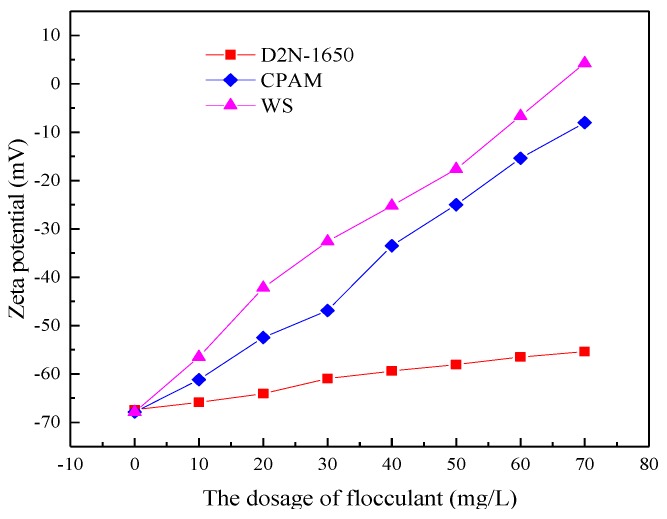
The effect of flocculant on Zeta potential.

The simulated ASP flooding produced water with 400 mg/L of polymer, 800 mg/L of NaOH and 200 mg/L of the surfactant was prepared. The flocculant WS was added into the produced water and both were settled in a water bath at a temperature of 45 °C for 4 h to study the effect of the flocculant on Zeta potential for the ASP flooding produced water. The effects of CPAM and D2N-1650 on Zeta potential were studied respectively under the same experimental conditions, as shown in Figure 6. The flocculant CPAM and D2N-1650 can reduce the absolute value of the Zeta potential individually, however the interaction of the two flocculants can reduce the absolute value of Zeta potential even further. A large number of positive charges on the molecular chain from the flocculant CPAM neutralized the negative charge on the surface of the oil droplet. Then the absolute value of Zeta potential on the droplet surface decreased greatly. The flocculant D2N-1650 did not have positive charges. It can only bind the oil droplets together to produce precipitation through “adsorption bridging”. Therefore, the influence of the flocculant D2N-1650 on the Zeta potential was limited.

### 3.6. The Effect of Flocculant on the Size of Oil Droplets

**Figure 7:**
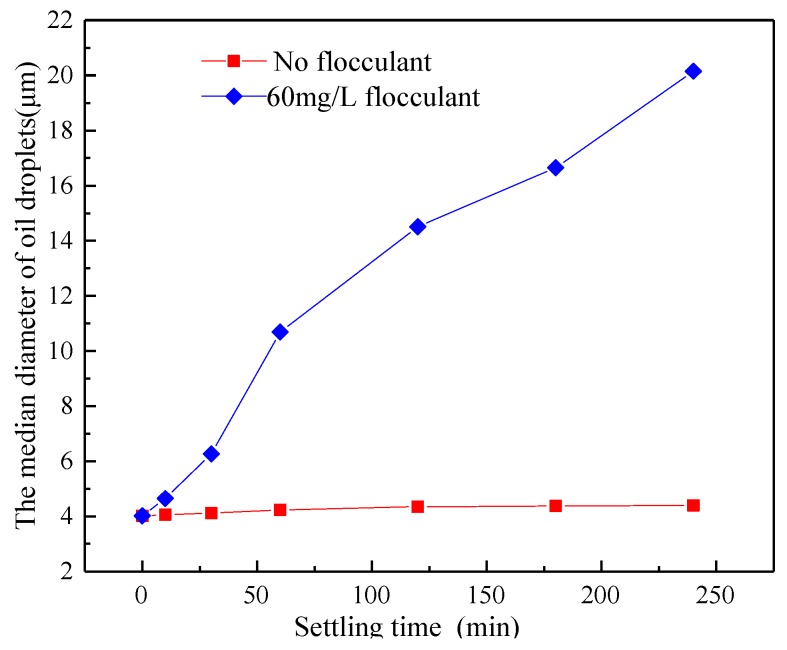
The effect of flocculant on the median diameter of oil droplets.

**Figure 8:**
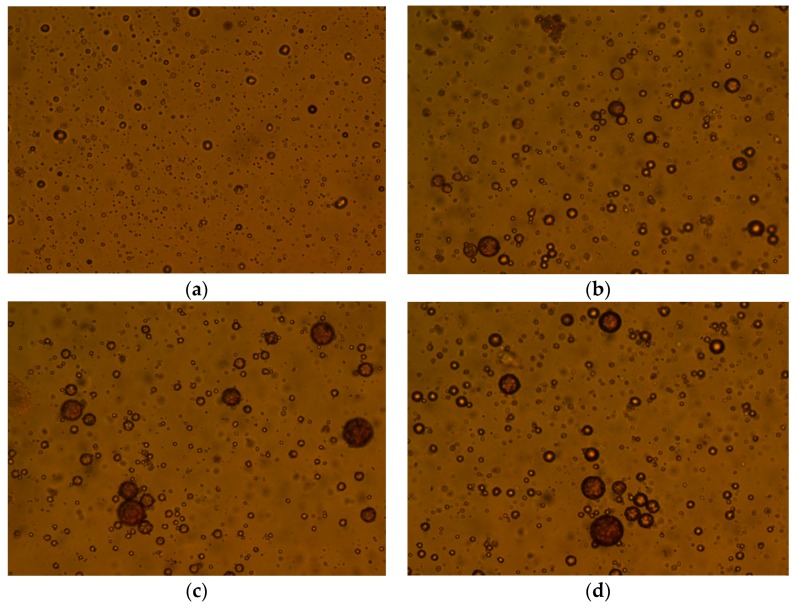
The effect of flocculant on the size of oil droplet. (**a**) Settlement without flocculant for 0 min, (**b**) settlement without flocculant for 120 min, (**c**) settlement with flocculant for 0 min, (**d**) settlement with flocculant for 120 min.

The simulated ASP flooding produced water with 400 mg/L of polymer, 800 mg/L of NaOH and 200 mg/L of the surfactant was prepared. Then, 60 mg/L flocculant WS was added into the produced water and settled in a 45 °C water bath for 2 h to study the effect of flocculant on oil droplet median diameter, as shown in Figure 7 and Figure 8. The magnification used to produce Figure 8 was 10 × 40. There is an obvious effect of the flocculant increasing the median diameter of oil droplets. The median diameter of oil droplets increased when settled for 2 h without flocculant. The limited increase indicated that the ASP flooding produced water was very stable. However, the median diameter of oil droplets increased from 4.02 μm to 20.15 μm when settled for 2 h with added flocculant into the system. The significant increase of the median diameter of oil droplets indicated that the oil droplets in the ASP flooding produced water had become unstable after adding flocculant. However, when compared with adding demulsifier ZY, the effect of flocculant WS was relatively small. In order to further investigate the effect of demulsifier on the size of oil droplets, the shapes of oil droplets in the simulated produced water at different conditions were studied with a microscope, the results of which are shown in Figure 8. The size of oil droplets in the simulated produced water without flocculant was basically unchanged after settlement for 2 h. The size of oil droplets increased significantly after adding flocculant for settlement for 60 min. With an increase in settling time, the size of oil droplets increased gradually.

## 4. Demulsification-Flocculation Mechanism of Oil-Water Emulsion

**Figure 9:**
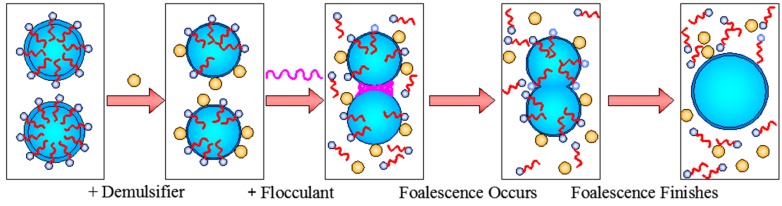
Demulsification–Flocculation mechanism.

After comparison and analysis of the theoretical and experimental results, the mechanism of demulsification and flocculation was summarized, as shown in Figure 9. The demulsifier molecules can be adsorbed onto the oil droplets, replacing the surfactant absorbed on the surface of oil droplet, to reduce the interfacial tension, and weaken interfacial film strength, so that the stability of oil droplets decreased. At the same time, the demulsifier can also neutralize the negative charge on the surface of the oil droplet, reducing the electrostatic repulsion between oil droplets, allowing the accumulation of oil droplets. The flocculant after demulsification of oil droplets by charge neutralization, adsorption bridging and sweep function together, so that the oil droplets form aggregates, then the synthetic action of the demulsifier and the flocculant causes the oil droplet film to break up and oil droplet coalescence occurs, so as to achieve the purpose of oil-water separation.

## 5. Conclusion

In this paper, we studied the demulsification-flocculation mechanism of produced water from ASP flooding by considering oil-water interfacial tension, Zeta potential and the size of oil droplets of produced water with added demulsifier and flocculent through laboratory experiments.

We demonstrated that the demulsification-flocculation mechanism is feasible and can be applied to the produced water from ASP flooding. The demulsifier molecules can be adsorbed onto the oil droplets and replace the surfactant absorbed on the surface of oil droplets, thereby reducing the interfacial tension and weakening the interfacial film strength. The demulsifier can also neutralize the negative charge on the surface of oil droplets and reduce the electrostatic repulsion between them, which is beneficial in the accumulation of oil droplets. The flocculent after demulsification of oil droplets by charge neutralization, adsorption bridging, and sweep function together. Therefore, the oil droplets form aggregates and the synthetic action of the demulsifier. The flocculent causes the oil drop film to break and then the oil droplets coalesce, resulting in oil water separation.

## Figures and Tables

**Table 1:** Experimental drugs and sources.

Drugs	Sources
NaCl	Tianjin Damao Chemical Plant
Na_2_HCO_3_	Tianjin Yaohua Chemical Plant
Na_2_CO_3_	Tianjin Yaohua Chemical Plant
Na_2_SO_4_	Tianjin Yaohua Chemical Plant
CaCl_2_	Harbin Xinda Chemical Plant
MgCl_2_	Tianjin Fuchen Chemical Plant
Petroleum Ether	Shenyang W&S Chemical Plant

All of the drugs above are analytical reagents.

**Table 2:** Experimental instruments.

Purpose	Instrument Name	Types	Manufacturer
Preparation of emulsion	Digital display disperser	IKAT25	IKA Company
Zeta potential	Micro electrophoresis apparatus	JS94H	Shanghai Zhongchen Digital technology equipment Co. Ltd.
Oil droplet size distribution	Laser particle size analyzer	BT-9300H	Dandong Better Science and Technology Co. Ltd.
Interfacial tension	Interface tension meter	XZD-5	Beijing Hake Experimental Instrument Factory
Temperature control	Thermostatic water bath	S501-2	Liaoyang Huaguang Instrument Factory
Micrographs observation	Biological microscope	IX73	Shanghai Puhe Biotechnology Co. Ltd.
Weigh	Electronic balance	BS210S	Sartorius scientific Instruments Co. Ltd.
Quantitative transfer liquid	Micropipette	Eppendorf	Eppendorf China Co. Ltd.
